# Crystal structure of zwitterionic 3-(2-hy­droxy-2-phospho­nato-2-phosphono­eth­yl)imidazo[1,2-*a*]pyridin-1-ium monohydrate (minodronic acid monohydrate): a redetermination

**DOI:** 10.1107/S2056989014026863

**Published:** 2015-01-01

**Authors:** Annalisa Airoldi, Piergiorgio Bettoni, Monica Donnola, Gianluca Calestani, Corrado Rizzoli

**Affiliations:** a R&D Division, PROCOS S.p.A., Via G. Matteotti 249, 28062 Cameri (Novara), Italy; b Universitá degli Studi di Parma, Dipartimento di Chimica, Parco Area delle Scienze 17/A, 43124 Parma, Italy

**Keywords:** crystal structure, bis­phospho­nate, polymorphism, minodronic acid, hydrogen bonds, redetermination

## Abstract

In a previous study, the X-ray structure of the title compound, C_9_H_12_N_2_O_7_P_2_·H_2_O, was reported [Takeuchi *et al.*, (1998 ▸). *Chem. Pharm. Bull.* **46**, 1703–1709], but neither atomic coordinates nor details of the geometry were published. The structure has been redetermined with high precision as its detailed knowledge is essential to elucidate the presumed polymorphism of minodronic acid monohydrate at room temperature. The mol­ecule crystallizes in a zwitterionic form with cationic imidazolium[1,2*a*]pyridine and anionic phospho­nate groups. The dihedral angle formed by the planes of the pyridine and imidazole rings is 3.55 (9)°. A short intra­molecular C—H⋯O contact is present. In the crystal, mol­ecules are linked by O—H⋯O, N—H⋯O and C—H⋯O hydrogen bonds and π–π inter­actions [centroid-to-centroid distance = 3.5822 (11) Å], forming a three-dimensional structure.

## Abstract 2

A redetermination of the crystal structure of minodronic acid monohydrate was carried out in order to provide accurate atomic coordinates and geometry information, whose knowledge is fundamental to elucidate the presumed polymorphism of the compound at room temperature.

## Chemical context   

Minodronic acid, (1-hy­droxy-2-(imidazo[1,2-a]pyridin-3-yl)ethane-1,1-bis­(phospho­nic acid), has excellent bone resorption inhibitory activity, as well as anti-inflammatory, analgesic and anti­pyretic activities and is useful for the treatment of diseases in which an increased bone resorption participates (Tanishima & Morio, 2013 ▸; Yamane *et al.*, 2003 ▸; Ito *et al.*, 2010 ▸; Sato *et al.*, 2006 ▸). For practical uses and solid pharmaceutical preparations, the monohydrate form is preferred, giving more stable crystals with respect to the anhydrous and polyhydrate forms. By developing a pharmaceutical preparation (Takeuchi *et al.*, 1993 ▸), it was found that monohydrate crystals include two crystal forms, named *D* and *E*, having the same powder X-ray diffraction pattern but different dehydration temperatures. In fact, according to TG–DSC thermogravimetric analyses, crystal *D* has a dehydration peak temperature of 408 to 422 K, whereas for *E* it is 433 to 443 K. 
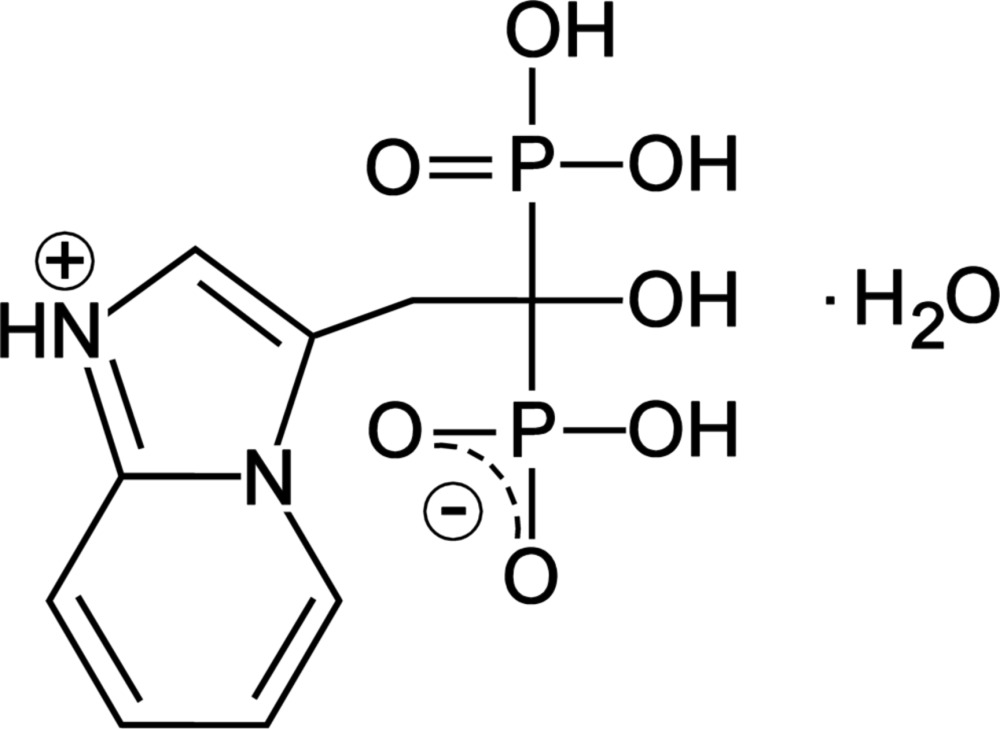


The monohydrate crystals of *D* and *E* are both produced by recrystallization of the free acid from aqueous hydro­chloric acid solutions by gradual cooling of a heat-dissolved solution under mild stirring conditions, followed by drying the crystals at 313–333 K under reduced pressure. The stirring mode and cooling conditions are the key factors in determining the final crystal form.

The relation between the two crystal forms deviates from the conventional concept of polymorphism by the similarity of the powder X-ray diffraction patterns. In principle, different mechanisms could be invoked to justify the different thermal behavior, amongst which a small difference in the crystal packing or in the atomic inter­actions could lead to it. The crystal structure of a monohydrate form of minodronic acid has been published earlier (Takeuchi *et al.*, 1998 ▸), but neither the atomic coordinates nor details of the mol­ecular and crystal geometry were reported. In the absence of detailed information, the present study of the crystal structures of both forms, *D* and *E*, was undertaken with the aim of finding a reasonable solution to the unusual ‘polymorphism’ problem. Batches of the *D* and *E* crystals were prepared as described in the *Experimental* section and characterized by thermogravimetric analysis.

**Figure 1:**
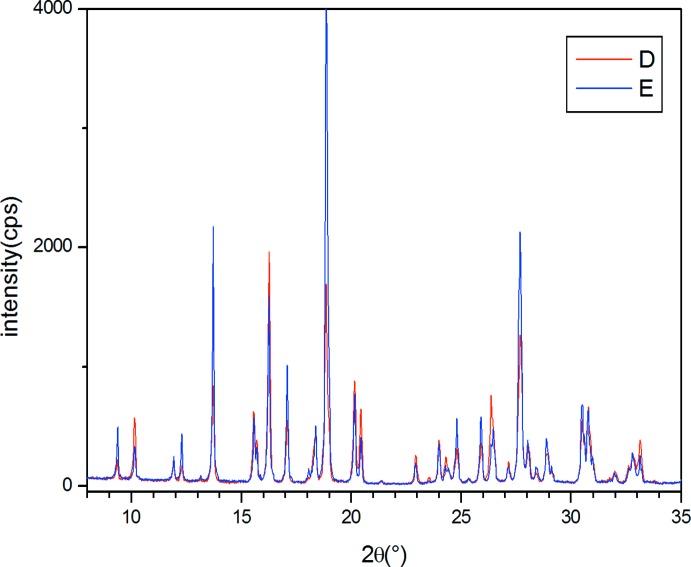
Comparison of the room-temperature powder X-ray diffraction patterns of forms *D* (red line) and *E* (blue line) of the title compound.

Powder X-ray diffraction was performed with a Thermo X’tra Diffractometer equipped with a Si(Li) solid state detector directly on the as-prepared samples without grinding (Fig. 1 ▸). This confirmed that the diffraction patterns of the two forms involve peaks occurring at the same 2θ angles, the unique difference consisting of a different intensity distribution probably originating from preferential orientation. It is noteworthy that both diffraction patterns are fully compatible with the lattice parameters reported by Takeuchi *et al.* (1998 ▸). Small single crystals suitable for XRD experiments were selected from both batches and their structures solved and refined. The analyses established that the crystal structures are perfectly comparable within experimental error, and compatible with that previously reported.

We report herein the redetermination of the crystal structure of form *D* of minodronic acid monohydrate, whereas that of form *E* has been deposited at the CCDC (Rizzoli & Calestani, 2014 ▸). On the basis of the present study, the peculiar thermal behaviour of the *D* and *E* forms of minodronic acid cannot be ascribed to structural differences, but it is probably due to morphological or microstructural features induced by the crystallization procedure and the influence of the dehydration process.

## Structural commentary   

**Figure 2:**
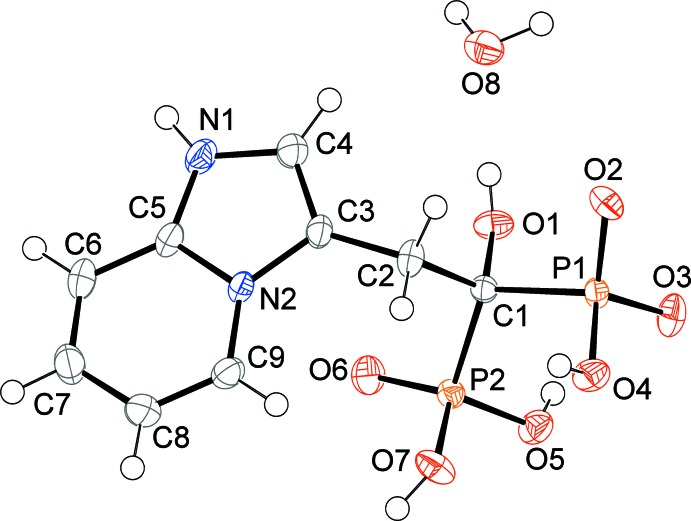
The mol­ecular structure of the title compound, showing the atom labelling. Displacement ellipsoids are drawn at the 50% probability level.

The asymmetric unit of the title compound, Fig. 2 ▸, consists of minodronic acid and a water mol­ecule of crystallization. The acid mol­ecule crystallizes in a zwitterionic form with cationic imidazolium[1,2a]pyridine and anionic phospho­nate groups. The fused-ring system deviates from planarity, with the dihedral angle formed by the pyridine and imidazole rings being 3.55 (9)°. An analysis of the bond lengths within the fused-ring system, indicates that the C=N and C=C double-bond distribution shown in the Scheme is the most probable, but a resonant form involving the aromatic character of the pyridine ring and the localization of the positive change on the atom N2 also exists. The values of the P—O bond lengths indicate that the negative charge is delocalized on atoms O2 and O3, whose distances [P1—O2 = 1.5197 (10), P1—O3 = 1.5001 (10) Å] are inter­mediate between those observed for the protonated atoms O4, O5 and O7 [mean value 1.564 (7) Å] and for the localized P=O double bond [P2—O6 = 1.4817 (10) Å]. The observed distribution of the hydrogen atoms and of the charge on the phospho­nate groups differs from that deducible from the mol­ecular plot reported previously by Takeuchi *et al.* (1998 ▸). An intra­molecular C—H⋯O hydrogen bond is present (Table 1 ▸).

## Supra­molecular features   

**Figure 3:**
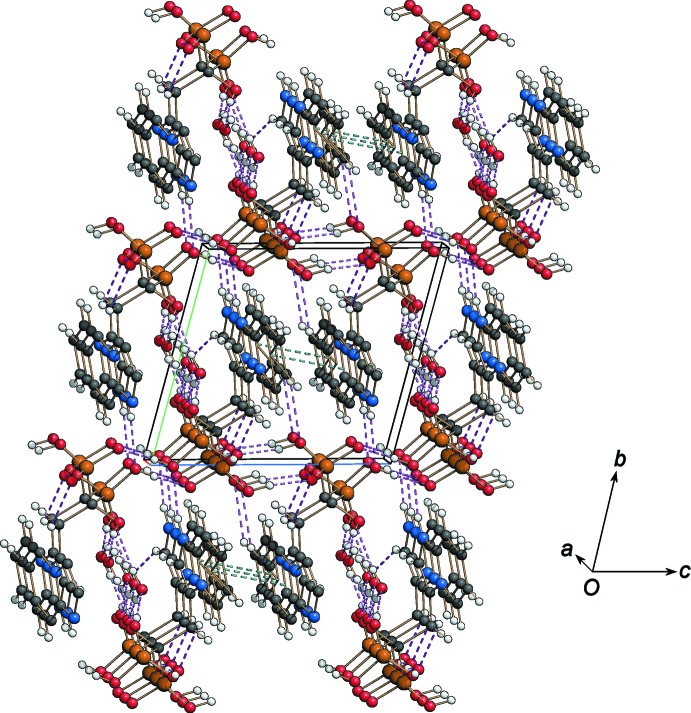
The crystal packing of the title compound viewed along the *a* axis, showing the hydrogen-bonding network (violet dashed lines) and π–π inter­actions (blue dashed lines); see Table 1 ▸ for details.

**Figure 4:**
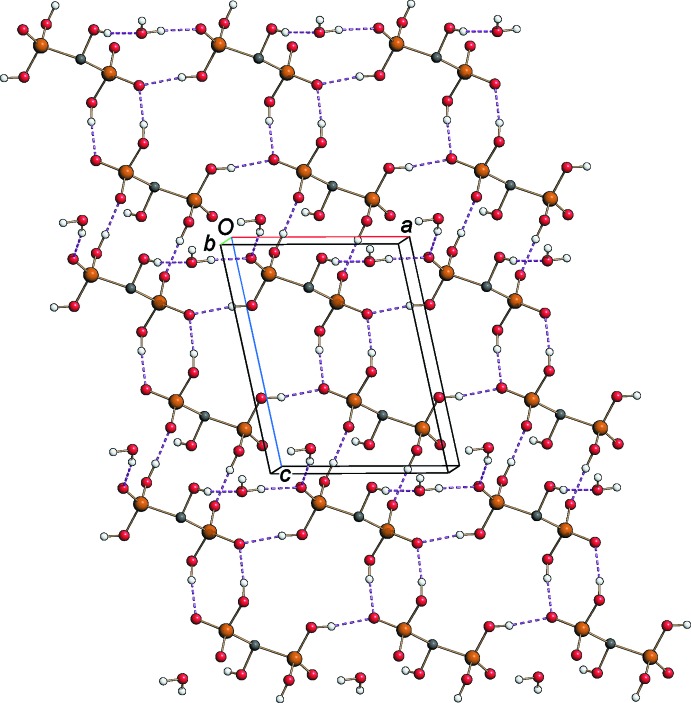
A view along the *b* axis of the hydrogen-bonding network (dashed lines) in the phospho­nate layer (see Table 1 ▸ for detail). Nitro­gen, carbon (except C1) and C- and N-bound H atoms have been omitted for clarity.

The crystal structure of the title compound is characterized by a very effective hydrogen-bonding network which is responsible for the unusually high value of the calculated density of the crystal (1.818 g cm^−3^). As shown in Fig. 3 ▸, the crystal packing may be described as an alternate stacking along the *b* axis of phospho­nate and organic layers forming a three-dimensional network through O—H⋯O, N—H⋯O and C—H⋯O hydrogen bonds (Table 1 ▸ and Fig. 3 ▸). Adjacent phospho­nate layers are bridged *via* hydrogen bonds involving the water mol­ecules, which are hosted inside channels parallel to the *a* axis and running between pairs of fused-ring systems connected by π–π inter­actions [*Cg*1⋯*Cg*1^i^ = 3.5822 (11) Å; *Cg*1 is the centroid of the N2/C5–C9 ring; symmetry code: (i) −*x*, −y + 1, −*z* + 1]. The O—H⋯O hydrogen-bonding system within the phospho­nate layer (Fig. 4 ▸) generates rings arranged in 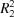
(8), 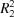
(12), 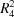
(16) and 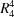
(16) graph-set motifs (Bernstein *et al.*, 1995 ▸).

## Synthesis and crystallization   

Crystal form *D*: crystals of minodronic acid (150 g) were added to 5.6 l of 1 *N* hydro­chloric acid (about 37.5 volumes) in a reactor equipped with mechanical stirring, and dissolved with heating. The dissolution temperature (368 K) was maintained for at least 30 min, followed by filtration of a small amount of undissolved particles. The clear filtrate was stirred at 110 r.p.m. and slowly cooled to 328 K in 4 h, and then to 295 K overnight. The crystalline precipitate was collected by filtration, washed with 300 ml of water and 300 ml of ethanol, and dried at 318 K to obtain 135 g of pure form *D* of minodronic acid monohydrate, as shown by the dehydration peak at 415 K (Takeuchi *et al.*, 1993 ▸). The colourless crystal sample used for X-ray analysis was selected from the batch.

Crystal form *E*: crystals of minodronic acid (15 g) were added to 0.6 l of 1 *N* hydro­chloric acid (about 40 volumes) in a reactor equipped with magnetic stirring and dissolved with heating. The dissolution temperature (368 K) was maintained for at least 30 min followed by filtration of a small amount of undissolved particles. The clear filtrate was stirred slowly so that the liquid surface could not create a vortex (<110 r.p.m.) and gradually cooled down to 295 K overnight. The crystal precipitate was collected by filtration, washed with 30 ml of water and 30 ml of ethanol, and dried at 318 K to obtain 14 g of pure *E* form of minodronic acid monohydrate, as shown by the dehydration peak at 438 K (Takeuchi *et al.*, 1993 ▸). The colourless crystal sample used for X-ray analysis was selected from the batch.

## Refinement   

Crystal data, data collection and structure refinement details are summarized in Table 2 ▸. All the H atoms were located in a difference Fourier map. The N- and O-bound hydrogen atoms were freely refined. The C-bound H atoms were refined using a riding model approximation, with C—H = 0.93–0.97 Å and with *U*_iso_(H) = 1.2*U*_eq_(C).

## Supplementary Material

Crystal structure: contains datablock(s) I. DOI: 10.1107/S2056989014026863/su5037sup1.cif

Structure factors: contains datablock(s) I. DOI: 10.1107/S2056989014026863/su5037Isup2.hkl

Supporting information file. DOI: 10.1107/S2056989014026863/su5037Isup3.cml

CCDC reference: 1038087

Additional supporting information:  crystallographic information; 3D view; checkCIF report

## Figures and Tables

**Table 1:** Hydrogen-bond geometry (, )

*D*H*A*	*D*H	H*A*	*D* *A*	*D*H*A*
O1H1*O*O8	0.78(3)	1.87(3)	2.6468(18)	169(2)
O4H4*O*O2^i^	0.90(3)	1.73(3)	2.6116(16)	169(3)
O5H5*O*O3^ii^	0.88(3)	1.62(3)	2.4973(14)	175(3)
O7H7*O*O2^iii^	0.83(2)	1.80(2)	2.6183(14)	170(2)
O8H81O6^iv^	0.82(2)	1.99(2)	2.8011(15)	169(3)
O8H82O6^v^	0.91(2)	1.83(2)	2.7334(16)	175(2)
N1H1*N*O5^vi^	0.85(2)	2.18(3)	2.9930(15)	158(2)
C2H2*B*O2	0.97	2.50	3.0224(17)	114
C4H4O8	0.93	2.40	3.2079(19)	145
C8H8O4^vii^	0.93	2.51	3.1704(17)	128

Symmetry codes: (i) 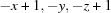; (ii) 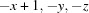; (iii) 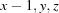; (iv) 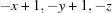; (v) 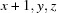; (vi) 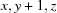; (vii) 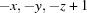.

**Table 2:** Experimental details

Crystal data	
Chemical formula	C_9_H_12_N_2_O_7_P_2_H_2_O
*M*_r_	340.16
Crystal system, space group	Triclinic, *P* 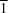
Temperature (K)	294
*a*, *b*, *c* ()	7.3668(11), 8.9833(12), 9.9733(18)
, , ()	75.0136(17), 77.2716(17), 88.5706(18)
*V* (^3^)	621.54(17)
*Z*	2
Radiation type	Mo *K*
(mm^1^)	0.40
Crystal size (mm)	0.18 0.15 0.10

Data collection	
Diffractometer	Bruker SMART 1000 CCD
Absorption correction	Multi-scan (*SADABS*; Bruker, 2008 ▸)
*T*_min_, *T*_max_	0.640, 0.746
No. of measured, independent and observed [*I* > 2(*I*)] reflections	10459, 4054, 3413
*R*_int_	0.020
(sin /)_max_ (^1^)	0.752

Refinement	
*R*[*F* ^2^ > 2(*F* ^2^)], *wR*(*F* ^2^), *S*	0.035, 0.101, 1.07
No. of reflections	4054
No. of parameters	218
H-atom treatment	H atoms treated by a mixture of independent and constrained refinement
_max_, _min_ (e ^3^)	0.55, 0.28

Computer programs: *APEX2* and *SAINT* (Bruker, 2008 ▸), *SIR97* (Altomare *et al.*, 1999 ▸), *SHELXL2014* (Sheldrick, 2008 ▸), *ORTEP-3 for Windows* (Farrugia, 2012 ▸), *SCHAKAL99* (Keller, 1999 ▸) and *publCIF* (Westrip, 2010 ▸).

## supplementary crystallographic information

### Crystal data

**Table:**

C_9_H_12_N_2_O_7_P_2_·H_2_O	*Z* = 2
*M**_r_* = 340.16	*F*(000) = 352
Triclinic, *P*1	*D*_x_ = 1.818 Mg m^−^^3^
*a* = 7.3668 (11) Å	Mo *K*α radiation, λ = 0.71073 Å
*b* = 8.9833 (12) Å	Cell parameters from 227 reflections
*c* = 9.9733 (18) Å	θ = 8.2–21.3°
α = 75.0136 (17)°	µ = 0.40 mm^−^^1^
β = 77.2716 (17)°	*T* = 294 K
γ = 88.5706 (18)°	Block, colourless
*V* = 621.54 (17) Å^3^	0.18 × 0.15 × 0.10 mm

### Data collection

**Table:**

Bruker SMART 1000 CCD diffractometer	3413 reflections with *I* > 2σ(*I*)
ω scan	*R*_int_ = 0.020
Absorption correction: multi-scan (*SADABS*; Bruker, 2008)	θ_max_ = 32.3°, θ_min_ = 2.2°
*T*_min_ = 0.640, *T*_max_ = 0.746	*h* = −11→10
10459 measured reflections	*k* = −12→13
4054 independent reflections	*l* = −14→14

### Refinement

**Table:**

Refinement on *F*^2^	0 restraints
Least-squares matrix: full	Hydrogen site location: mixed
*R*[*F*^2^ > 2σ(*F*^2^)] = 0.035	H atoms treated by a mixture of independent and constrained refinement
*wR*(*F*^2^) = 0.101	*w* = 1/[σ^2^(*F*_o_^2^) + (0.060*P*)^2^ + 0.1062*P*] where *P* = (*F*_o_^2^ + 2*F*_c_^2^)/3
*S* = 1.07	(Δ/σ)_max_ = 0.001
4054 reflections	Δρ_max_ = 0.55 e Å^−^^3^
218 parameters	Δρ_min_ = −0.28 e Å^−^^3^

### Special details

**Table:**

Geometry. All e.s.d.'s (except the e.s.d. in the dihedral angle between two l.s. planes) are estimated using the full covariance matrix. The cell e.s.d.'s are taken into account individually in the estimation of e.s.d.'s in distances, angles and torsion angles; correlations between e.s.d.'s in cell parameters are only used when they are defined by crystal symmetry. An approximate (isotropic) treatment of cell e.s.d.'s is used for estimating e.s.d.'s involving l.s. planes.

### Fractional atomic coordinates and isotropic or equivalent isotropic displacement parameters (Å^2^)

**Table:**

	*x*	*y*	*z*	*U*_iso_*/*U*_eq_	
P1	0.51328 (4)	−0.00236 (4)	0.28423 (3)	0.01430 (9)	
P2	0.17313 (4)	0.13064 (4)	0.15681 (3)	0.01514 (9)	
O1	0.49943 (14)	0.25308 (12)	0.08484 (10)	0.0206 (2)	
H1O	0.576 (3)	0.303 (3)	0.098 (2)	0.039 (6)*	
O2	0.67125 (13)	0.05492 (12)	0.33555 (10)	0.0208 (2)	
O3	0.56833 (15)	−0.09174 (12)	0.17446 (11)	0.0232 (2)	
O4	0.37247 (14)	−0.10822 (11)	0.41416 (11)	0.0214 (2)	
H4O	0.349 (4)	−0.080 (3)	0.496 (3)	0.070 (8)*	
O5	0.22204 (14)	0.01195 (11)	0.06740 (11)	0.0213 (2)	
H5O	0.298 (4)	0.044 (3)	−0.016 (3)	0.072 (8)*	
O6	0.10307 (14)	0.27806 (12)	0.08260 (12)	0.0258 (2)	
O7	0.03415 (14)	0.04399 (13)	0.29628 (11)	0.0240 (2)	
H7O	−0.079 (3)	0.054 (3)	0.300 (3)	0.050 (7)*	
O8	0.77583 (16)	0.42957 (14)	0.09502 (14)	0.0301 (3)	
H81	0.800 (3)	0.515 (3)	0.038 (3)	0.049 (7)*	
H82	0.884 (3)	0.379 (3)	0.086 (2)	0.049 (6)*	
N1	0.25482 (18)	0.67430 (14)	0.19284 (13)	0.0225 (2)	
H1N	0.277 (3)	0.769 (3)	0.148 (2)	0.047 (6)*	
N2	0.09082 (15)	0.46468 (12)	0.31543 (12)	0.0158 (2)	
C1	0.38347 (17)	0.16943 (14)	0.21539 (13)	0.0138 (2)	
C2	0.34325 (19)	0.26352 (15)	0.32898 (14)	0.0189 (2)	
H2A	0.2507	0.2061	0.4098	0.023*	
H2B	0.4565	0.2705	0.3619	0.023*	
C3	0.27645 (18)	0.42267 (15)	0.28236 (14)	0.0176 (2)	
C4	0.3748 (2)	0.55521 (16)	0.20766 (15)	0.0219 (3)	
H4	0.5030	0.5637	0.1723	0.026*	
C5	0.08150 (19)	0.62025 (15)	0.25700 (14)	0.0182 (2)	
C6	−0.0888 (2)	0.69424 (16)	0.27288 (16)	0.0236 (3)	
H6	−0.0965	0.7984	0.2295	0.028*	
C7	−0.2424 (2)	0.60818 (17)	0.35401 (17)	0.0260 (3)	
H7	−0.3572	0.6541	0.3670	0.031*	
C8	−0.2293 (2)	0.44988 (17)	0.41874 (16)	0.0240 (3)	
H8	−0.3350	0.3937	0.4766	0.029*	
C9	−0.06486 (19)	0.37863 (15)	0.39786 (15)	0.0200 (3)	
H9	−0.0576	0.2737	0.4385	0.024*	

### Atomic displacement parameters (Å^2^)

**Table:**

	*U*^11^	*U*^22^	*U*^33^	*U*^12^	*U*^13^	*U*^23^
P1	0.01367 (15)	0.01350 (15)	0.01500 (15)	0.00313 (11)	−0.00231 (11)	−0.00340 (11)
P2	0.01371 (15)	0.01476 (16)	0.01648 (16)	0.00114 (11)	−0.00367 (11)	−0.00301 (11)
O1	0.0207 (5)	0.0207 (5)	0.0165 (4)	−0.0069 (4)	−0.0002 (4)	−0.0004 (4)
O2	0.0134 (4)	0.0274 (5)	0.0221 (5)	0.0026 (4)	−0.0045 (4)	−0.0072 (4)
O3	0.0304 (5)	0.0192 (5)	0.0202 (5)	0.0075 (4)	−0.0029 (4)	−0.0084 (4)
O4	0.0239 (5)	0.0183 (5)	0.0185 (4)	−0.0037 (4)	−0.0016 (4)	−0.0008 (4)
O5	0.0248 (5)	0.0190 (5)	0.0213 (5)	−0.0009 (4)	−0.0038 (4)	−0.0084 (4)
O6	0.0243 (5)	0.0199 (5)	0.0327 (6)	0.0059 (4)	−0.0123 (4)	−0.0016 (4)
O7	0.0138 (5)	0.0324 (6)	0.0216 (5)	−0.0010 (4)	−0.0005 (4)	−0.0024 (4)
O8	0.0204 (5)	0.0258 (6)	0.0404 (7)	−0.0019 (4)	−0.0044 (5)	−0.0036 (5)
N1	0.0259 (6)	0.0131 (5)	0.0244 (6)	−0.0003 (4)	0.0008 (5)	−0.0028 (4)
N2	0.0186 (5)	0.0112 (5)	0.0179 (5)	0.0024 (4)	−0.0037 (4)	−0.0044 (4)
C1	0.0132 (5)	0.0131 (5)	0.0142 (5)	0.0004 (4)	−0.0017 (4)	−0.0028 (4)
C2	0.0235 (6)	0.0156 (6)	0.0192 (6)	0.0063 (5)	−0.0070 (5)	−0.0060 (5)
C3	0.0188 (6)	0.0149 (6)	0.0198 (6)	0.0035 (4)	−0.0034 (5)	−0.0070 (5)
C4	0.0206 (6)	0.0193 (6)	0.0247 (7)	0.0010 (5)	−0.0007 (5)	−0.0077 (5)
C5	0.0236 (6)	0.0121 (5)	0.0183 (6)	0.0021 (4)	−0.0033 (5)	−0.0040 (4)
C6	0.0277 (7)	0.0164 (6)	0.0273 (7)	0.0077 (5)	−0.0080 (6)	−0.0059 (5)
C7	0.0221 (7)	0.0264 (7)	0.0324 (7)	0.0079 (5)	−0.0074 (6)	−0.0121 (6)
C8	0.0197 (6)	0.0240 (7)	0.0271 (7)	−0.0017 (5)	−0.0015 (5)	−0.0075 (5)
C9	0.0222 (6)	0.0153 (6)	0.0211 (6)	−0.0018 (5)	−0.0029 (5)	−0.0038 (5)

### Geometric parameters (Å, º)

**Table:**

P1—O3	1.5001 (10)	N2—C9	1.3743 (17)
P1—O2	1.5197 (10)	N2—C5	1.3744 (16)
P1—O4	1.5745 (10)	N2—C3	1.4004 (17)
P1—C1	1.8534 (13)	C1—C2	1.5558 (17)
P2—O6	1.4817 (10)	C2—C3	1.4914 (18)
P2—O5	1.5468 (10)	C2—H2A	0.9700
P2—O7	1.5625 (10)	C2—H2B	0.9700
P2—C1	1.8426 (13)	C3—C4	1.3580 (19)
O1—C1	1.4233 (15)	C4—H4	0.9300
O1—H1O	0.78 (2)	C5—C6	1.4022 (19)
O4—H4O	0.90 (3)	C6—C7	1.360 (2)
O5—H5O	0.88 (3)	C6—H6	0.9300
O7—H7O	0.83 (2)	C7—C8	1.412 (2)
O8—H81	0.83 (3)	C7—H7	0.9300
O8—H82	0.91 (2)	C8—C9	1.356 (2)
N1—C5	1.3363 (18)	C8—H8	0.9300
N1—C4	1.3700 (18)	C9—H9	0.9300
N1—H1N	0.85 (2)		
			
O3—P1—O2	116.20 (6)	P2—C1—P1	115.68 (7)
O3—P1—O4	107.20 (6)	C3—C2—C1	116.39 (11)
O2—P1—O4	109.85 (6)	C3—C2—H2A	108.2
O3—P1—C1	110.32 (6)	C1—C2—H2A	108.2
O2—P1—C1	106.68 (6)	C3—C2—H2B	108.2
O4—P1—C1	106.17 (6)	C1—C2—H2B	108.2
O6—P2—O5	115.07 (6)	H2A—C2—H2B	107.3
O6—P2—O7	113.46 (6)	C4—C3—N2	105.72 (11)
O5—P2—O7	105.64 (6)	C4—C3—C2	129.60 (13)
O6—P2—C1	109.47 (6)	N2—C3—C2	124.62 (12)
O5—P2—C1	107.81 (6)	C3—C4—N1	109.01 (13)
O7—P2—C1	104.75 (6)	C3—C4—H4	125.5
C1—O1—H1O	111.6 (16)	N1—C4—H4	125.5
P1—O4—H4O	116.9 (17)	N1—C5—N2	107.39 (11)
P2—O5—H5O	116.7 (18)	N1—C5—C6	131.56 (13)
P2—O7—H7O	118.0 (17)	N2—C5—C6	121.04 (12)
H81—O8—H82	105 (2)	C7—C6—C5	117.60 (13)
C5—N1—C4	109.26 (12)	C7—C6—H6	121.2
C5—N1—H1N	121.0 (15)	C5—C6—H6	121.2
C4—N1—H1N	129.6 (15)	C6—C7—C8	120.72 (13)
C9—N2—C5	120.93 (11)	C6—C7—H7	119.6
C9—N2—C3	130.37 (11)	C8—C7—H7	119.6
C5—N2—C3	108.59 (11)	C9—C8—C7	120.97 (13)
O1—C1—C2	112.48 (10)	C9—C8—H8	119.5
O1—C1—P2	101.50 (8)	C7—C8—H8	119.5
C2—C1—P2	112.69 (8)	C8—C9—N2	118.63 (12)
O1—C1—P1	106.38 (8)	C8—C9—H9	120.7
C2—C1—P1	107.91 (8)	N2—C9—H9	120.7

### Hydrogen-bond geometry (Å, º)

**Table:**

*D*—H···*A*	*D*—H	H···*A*	*D*···*A*	*D*—H···*A*
O1—H1*O*···O8	0.78 (3)	1.87 (3)	2.6468 (18)	169 (2)
O4—H4*O*···O2^i^	0.90 (3)	1.73 (3)	2.6116 (16)	169 (3)
O5—H5*O*···O3^ii^	0.88 (3)	1.62 (3)	2.4973 (14)	175 (3)
O7—H7*O*···O2^iii^	0.83 (2)	1.80 (2)	2.6183 (14)	170 (2)
O8—H81···O6^iv^	0.82 (2)	1.99 (2)	2.8011 (15)	169 (3)
O8—H82···O6^v^	0.91 (2)	1.83 (2)	2.7334 (16)	175 (2)
N1—H1*N*···O5^vi^	0.85 (2)	2.18 (3)	2.9930 (15)	158 (2)
C2—H2*B*···O2	0.97	2.50	3.0224 (17)	114
C4—H4···O8	0.93	2.40	3.2079 (19)	145
C8—H8···O4^vii^	0.93	2.51	3.1704 (17)	128

Symmetry codes: (i) −*x*+1, −*y*, −*z*+1; (ii) −*x*+1, −*y*, −*z*; (iii) *x*−1, *y*, *z*; (iv) −*x*+1, −*y*+1, −*z*; (v) *x*+1, *y*, *z*; (vi) *x*, *y*+1, *z*; (vii) −*x*, −*y*, −*z*+1.
